# A novel optical sensor system for the automatic classification of mosquitoes by genus and sex with high levels of accuracy

**DOI:** 10.1186/s13071-022-05324-5

**Published:** 2022-06-06

**Authors:** María I. González-Pérez, Bastian Faulhaber, Mark Williams, Josep Brosa, Carles Aranda, Nuria Pujol, Marta Verdún, Pancraç Villalonga, Joao Encarnação, Núria Busquets, Sandra Talavera

**Affiliations:** 1 IRTA, Centre de Recerca en Sanitat Animal (CReSA, IRTA-UAB), Campus de la Universitat Autònoma de Barcelona, Bellaterra, Cerdanyola del Vallès, Spain; 2 Irideon SL, Barcelona, Spain; 3 Servei de Control de Mosquits del Consell Comarcal del Baix Llobregat, Barcelona, Spain

**Keywords:** Mosquito trap, Automatic classification, Optical sensor, Machine learning, Deep learning, *Aedes*, *Culex*, Genus and sex classification, Mosquito surveillance

## Abstract

**Background:**

Every year, more than 700,000 people die from vector-borne diseases, mainly transmitted by mosquitoes. Vector surveillance plays a major role in the control of these diseases and requires accurate and rapid taxonomical identification. New approaches to mosquito surveillance include the use of acoustic and optical sensors in combination with machine learning techniques to provide an automatic classification of mosquitoes based on their flight characteristics, including wingbeat frequency. The development and application of these methods could enable the remote monitoring of mosquito populations in the field, which could lead to significant improvements in vector surveillance.

**Methods:**

A novel optical sensor prototype coupled to a commercial mosquito trap was tested in laboratory conditions for the automatic classification of mosquitoes by genus and sex. Recordings of > 4300 laboratory-reared mosquitoes of *Aedes* and *Culex* genera were made using the sensor. The chosen genera include mosquito species that have a major impact on public health in many parts of the world. Five features were extracted from each recording to form balanced datasets and used for the training and evaluation of five different machine learning algorithms to achieve the best model for mosquito classification.

**Results:**

The best accuracy results achieved using machine learning were: 94.2% for genus classification, 99.4% for sex classification of *Aedes*, and 100% for sex classification of *Culex*. The best algorithms and features were deep neural network with spectrogram for genus classification and gradient boosting with Mel Frequency Cepstrum Coefficients among others for sex classification of either genus.

**Conclusions:**

To our knowledge, this is the first time that a sensor coupled to a standard mosquito suction trap has provided automatic classification of mosquito genus and sex with high accuracy using a large number of unique samples with class balance. This system represents an improvement of the state of the art in mosquito surveillance and encourages future use of the sensor for remote, real-time characterization of mosquito populations.

**Graphical abstract:**

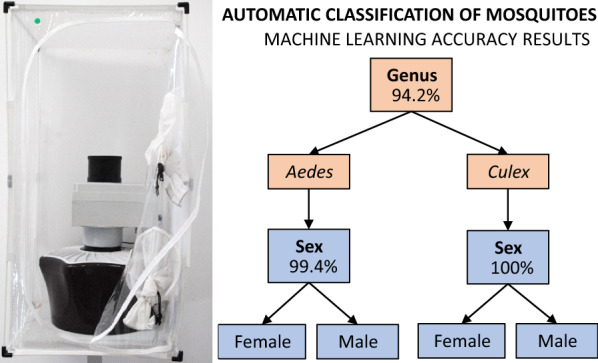

**Supplementary Information:**

The online version contains supplementary material available at 10.1186/s13071-022-05324-5.

## Background

Approximately 80% of the world’s human population lives with the risk of one or more vector-borne diseases (VBDs), and every year > 700,000 people die as a result [1]. In an increasingly connected world, travel and trade contribute to the spread of VBDs. Furthermore, a global warming scenario may lead to more favourable conditions for the survival and life cycle completion of the vectors [2] and may affect their abundance and distribution [3]. Mosquitoes (Diptera: Culicidae), particularly those belonging to *Aedes, Anopheles* and *Culex* genera, are one of the deadliest vectors worldwide. Mosquito species can transmit diseases such as malaria, dengue, yellow fever, West Nile fever, Zika, chikungunya and others [4]. According to World Health Organization directives [5] and European Centre for Disease Prevention and Control guidelines [6, 7], appropriate surveillance methods and indicators are needed to: determine the composition and monitor changes in mosquito populations, identify the presence of new invasive species, monitor mosquito-borne diseases, quantify the transmission potential of vectors and enable the design of accurate control programmes.

A range of insect trap types and methods are used in regular monitoring and surveillance of immature and/or adult mosquito populations [8]. Although immature stage monitoring can be easier to set up, it is not useful for estimating adult abundance due to the lack of correlation between egg, larval and pupal density indices and adult indices [9]. Studies show that the seasonal variation in mosquito abundance is better represented by adult trap monitoring than by other indices (e.g. House Index) based on immature stages [10]. Therefore, adult mosquito surveillance is generally the most widely applicable and accurate solution, especially for VBD risk assessment [11]. Many adult mosquito monitoring systems rely on traps using light, chemical attractants or CO_2_ as a bait. Most traps include a suction fan to draw approaching insects into a catch bag within the trap, and such types have been successfully used in many studies [12–14]. However, they require the catch bag to be periodically collected in the field, followed by a time-consuming process of identification of the collected specimens by entomologists. The time delay between insect trapping and analysis may limit the correct characterization of the temporal dynamics of mosquito populations. Such delays may also result in degradation of the insects in the catch bag because of desiccation or predation. New approaches to entomological surveillance include novel optical sensors to sense the characteristics of flying mosquitoes and analysis methods including machine learning methods to enable classification of mosquitoes in near real-time [15–19], which is crucial for surveillance programs.

Since the 1940s, microphones have been used to sense the audible flight tones emitted by flying mosquitoes, which may be associated with a particular mosquito genus, species or sex [20]. Acoustic methods are still employed today in applications such as sound traps, which emit species- and sex-specific sound frequencies to attract mosquitoes [21], and in classification systems such as those in which citizen scientists use their mobile phones to record mosquitoes [22, 23]. However, it is hard to obtain acceptable quality audio recordings of free-flying insects in the field because of the presence of background noise [18]. To address this, optical methods have been employed in which a light source is used to illuminate the flying insect and a light sensor is used to detect the light reflected and scattered, or attenuated, by the insect in flight [24–30]. The use of optical methods in this field began in 1955 when a photoelectric cell was used to detect the light modulation produced by a flying insect crossing its field of view [31]. In recent years, several optoelectronic sensors have been developed and used in conjunction with machine learning techniques to classify flying mosquitoes, with promising levels of accuracy [16, 17, 32–34].

Variables known to condition mosquito wingbeat fundamental frequency or its detection include taxonomy, sex, parity status, size, age, environmental temperature or wind speed [35–39]. Historically, wingbeat frequency has been used as the only predictor variable for mosquito classification, but it appears insufficient on its own to differentiate between mosquito species, especially those of the same genus [18]. This could limit field applications, where different mosquito species can coexist, with the possibility of overlap in wingbeat frequency distributions [40]. Efforts have been made in recent years to improve classification methods to distinguish among mosquito species, sex and even parity status [16, 17, 35]. In some cases, more advanced optical approaches have been used, for example to determine insect body and wing depolarization ratio, to improve the accuracy of classification [17].

In addition to the selection of the proper predictor variables and machine learning algorithms, the use of metadata such as the climatic conditions, the spatiotemporal localization and other ecological features accompanying mosquito captures may also be relevant for remote mosquito classification in the field [18, 33], since different mosquito species have different behaviour and ecological needs (e.g. geographical distribution, climatic range, circadian rhythm, and peaks of activity). According to new paradigms of remote mosquito surveillance, wingbeat sensor information and metadata could be sent wirelessly in real time to a server using *Internet of Things* (IoT) technology [41–43] with the potential to improve entomological surveillance.

Currently, there is only one commercial optical sensor product available for the remote monitoring of mosquito populations [41]. It is called the BG-Counter (Biogents, Germany), which according to the company, can distinguish mosquitoes from other insects and count mosquitoes. However, the sensor does not provide information about mosquito genus, species, sex or other attributes.

In this study, we present the results of a prototype optical sensor, which is coupled to the entrance of a commercial mosquito trap. The trap is of a type widely used for mosquito surveillance in the field and contains a suction fan. The fan causes the mosquitoes to pass through the sensor more quickly and with a more perturbed wingbeat compared to free flight conditions as described in another work [39]. For the present work, 4335 flights from mosquitoes of *Aedes* and *Culex* genera were recorded using the sensor. The three species for the study, *Aedes albopictus, Aedes aegypti* and *Culex pipiens*, were chosen because they are major vectors of arboviruses, have a significant impact on public health and are a focus of vector surveillance and control programs in many parts of the world. A set of features were extracted from each recording and used to train a series of machine learning algorithms to determine which combination of feature and algorithm gave the best performance in classifying mosquitoes by genus and sex. Whilst the scope of this work is limited to the classification of genus (*Aedes/Culex*) and sex (female/male), the inclusion of the two *Aedes* species in this study improves the genetic variability and permits future work on species classification using the data set from the present work.

## Methods

### Mosquito rearing conditions

As stated, three species of mosquitoes, from two genera, were used to generate the dataset:

i. *Aedes albopictus*, population of Sant Cugat del Vallès (2005), Barcelona, Spain (41.4667°, 2.0833°).
ii. *Aedes aegypti*, population of Paea (1994), Tahiti, French Polynesia (− 17.6889°, − 149.5869°).
iii. *Culex pipiens*, population of Gavà (2012), Barcelona, Spain (41.3000°, 2.0167°).

The mosquito populations were all reared under controlled environmental conditions in a climatic chamber at a temperature of 28 °C and a relative humidity of 80%, with a light:dark photoperiod of 12:12 h, except for *Cx. pipiens* (with a light:dark photoperiod of 11:11 h plus 1 h of dusk and 1 h of dawn). *Culex pipiens* and *Ae. albopictus* were reared in a biosafety level 2 (BSL2) laboratory and *Ae. aegypti* in a biosafety level 3 (BSL3) laboratory at IRTA-CReSA facilities. Larvae were maintained in plastic trays with 750 ml of dechlorinated tap water (renewed three times per week) and were fed with fish pellets (Goldfish Sticks-TETRA, Melle, Germany) ad libitum. Pupae, upon appearance, were immediately placed in insect cages (BugDorm-1 Insect Rearing Cage W30 × D30 × H30 cm, MegaView Science, Talchung, Taiwan). After metamorphosis, adults were fed with sucrose solution (10%) ad libitum. Females were not fed with blood to avoid any body size or flight variation. For *Aedes* females, the sucrose solution was removed 24 h before the sensor tests. For *Cx. pipiens* females, this was done 48 h before to improve their affinity for the attractant used in the trap.

### Sensor and trap description

**Fig. 1:**
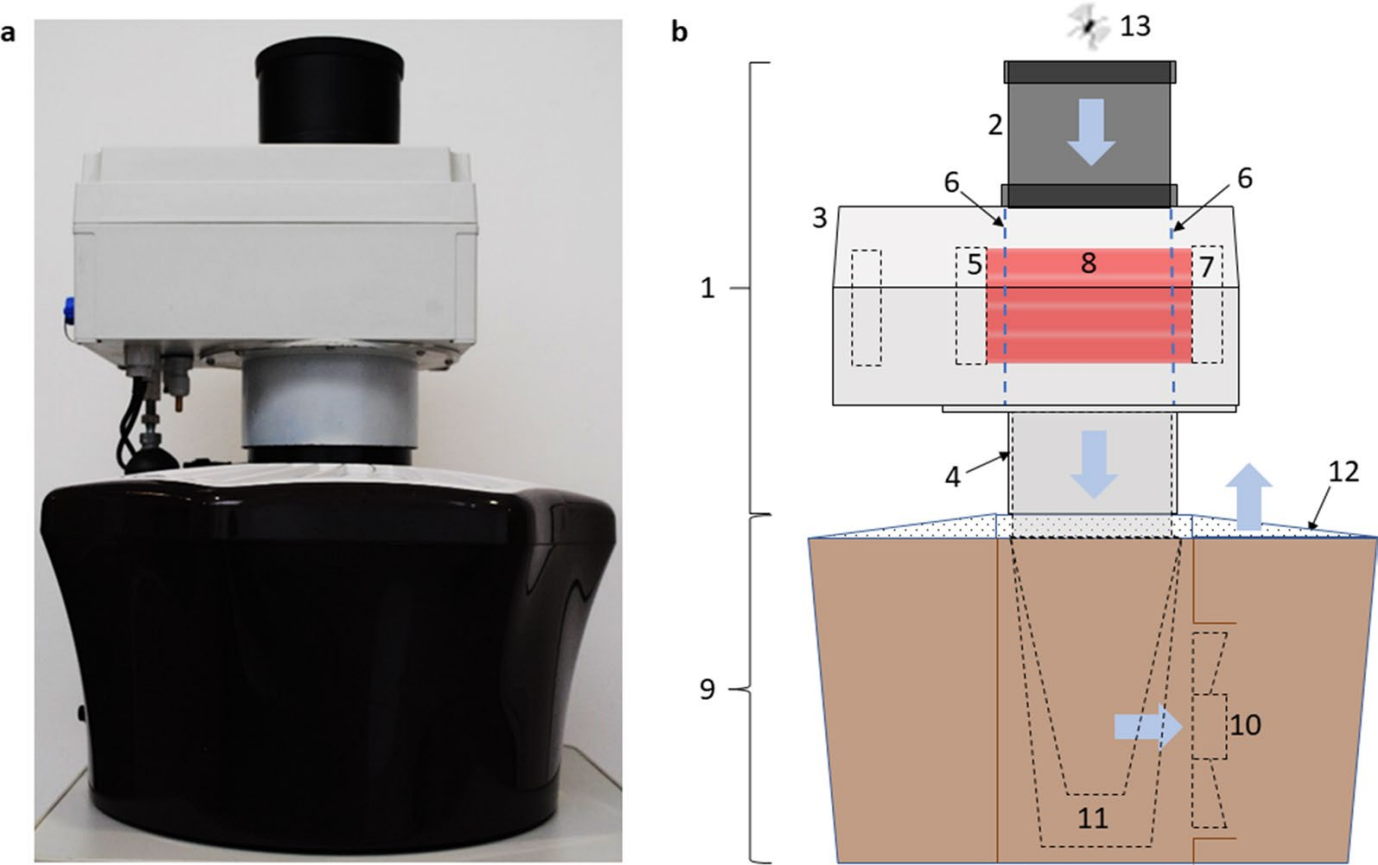
**a** Prototype sensor (top) fitted to a BG-Mosquitaire trap (bottom). **b** Side view diagram of sensor and trap to illustrate operation. The exterior of the sensor unit (1) is formed by an inlet tube with a diameter of approximately 100 mm (2), sensor housing (3) and outlet tube (4). The housing contains an optical emitter (5), which projects collimated beams of light through the transparent flight tube (6) and onto an optical receiver (7) to create a sensing zone (8) within the flight tube. The trap (9) contains a suction fan (10), a removable catch bag (11) made of textile mesh and a perforated lid (12). The fan produces a flow of air downward through the inlet tube, flight tube and catch bag and upward through the perforated lid as indicated by the blue arrows. An insect (13) which flies close to the entrance of the inlet tube may then be sucked downwards through the sensing zone where it will be recorded and then trapped in the catch bag. As the mosquito passes through the sensing zone it casts a shadow upon the optical receiver according to the so-called optical extinction mode of operation. As the insect flaps its wings within the sensing zone, the light falling on the optical receiver is modulated, giving rise to changes in the amplitude in the recorded waveform

The prototype sensor was designed and produced by Irideon SL (Barcelona, Spain) and was coupled to the entrance of a commercial BG-Mosquitaire suction trap from Biogents AG (Regensburg, Germany), as shown in Fig. 1a.

The trap coupled to the sensor was placed in an insect-rearing cage (BugDorm-4S4590 W47.5 × D47.5 × H93.0 cm, MegaView Science, Talchung, Taiwan). The trap was fitted with a sachet of BG-Sweetscent chemical attractant from Biogents AG. The air flow generated by the fan was approximately 3 m/s in the downward direction. When a mosquito flies close to the entrance funnel of the sensor, it may be sucked in by the fan, detected by the sensor and then trapped in the catch bag inside the body of the trap, as shown in Fig. 1b.

The sensor contains an optical emitter panel and an optical receiver panel, which face each other through a transparent flight tube with a diameter of 105 mm. The optical emitter comprises a two-dimensional (2D) array of 940-nm wavelength infrared light-emitting diodes (LEDs), and the optical receiver comprises a 2D array of 940-nm photodiodes. The optical sensor has an active length of 70 mm in the downward direction. These elements are also shown in Fig. 1b.

The output of the optical receiver is amplified and acquired by an analog to the digital converter (ADC) with a sampling frequency of 9603 samples per second. When a mosquito enters the sensing volume, it automatically triggers a recording of up to 1024 samples, i.e. of up to 107 ms duration. The duration of a typical mosquito flight is around 50 ms. The sensor automatically adds a timestamp to each recording, along with the measured ambient temperature.

### Data acquisition process

Mosquitoes from *Aedes* and *Culex* genera were anesthetized with carbon dioxide 48 and 72 h respectively before each experiment. They were separated into groups by species (*Cx. pipiens*, *Ae. albopictus* and *Ae. aegypti*) and sex (male, female).

*Culex pipiens* and *Ae. albopictus* were introduced into the insect rearing cage in batches of 20 individuals to reduce the chance of multiple mosquitoes passing through the sensor simultaneously. Batches of ten individuals were used for *Ae*. *aegypti* because of their greater affinity to the attractant. All mosquitoes were introduced at a distance of 20 to 30 cm from the entrance of the sensor to ensure that they could fly freely until they approached it and were sucked in to approximate field conditions.

**Fig. 2:**
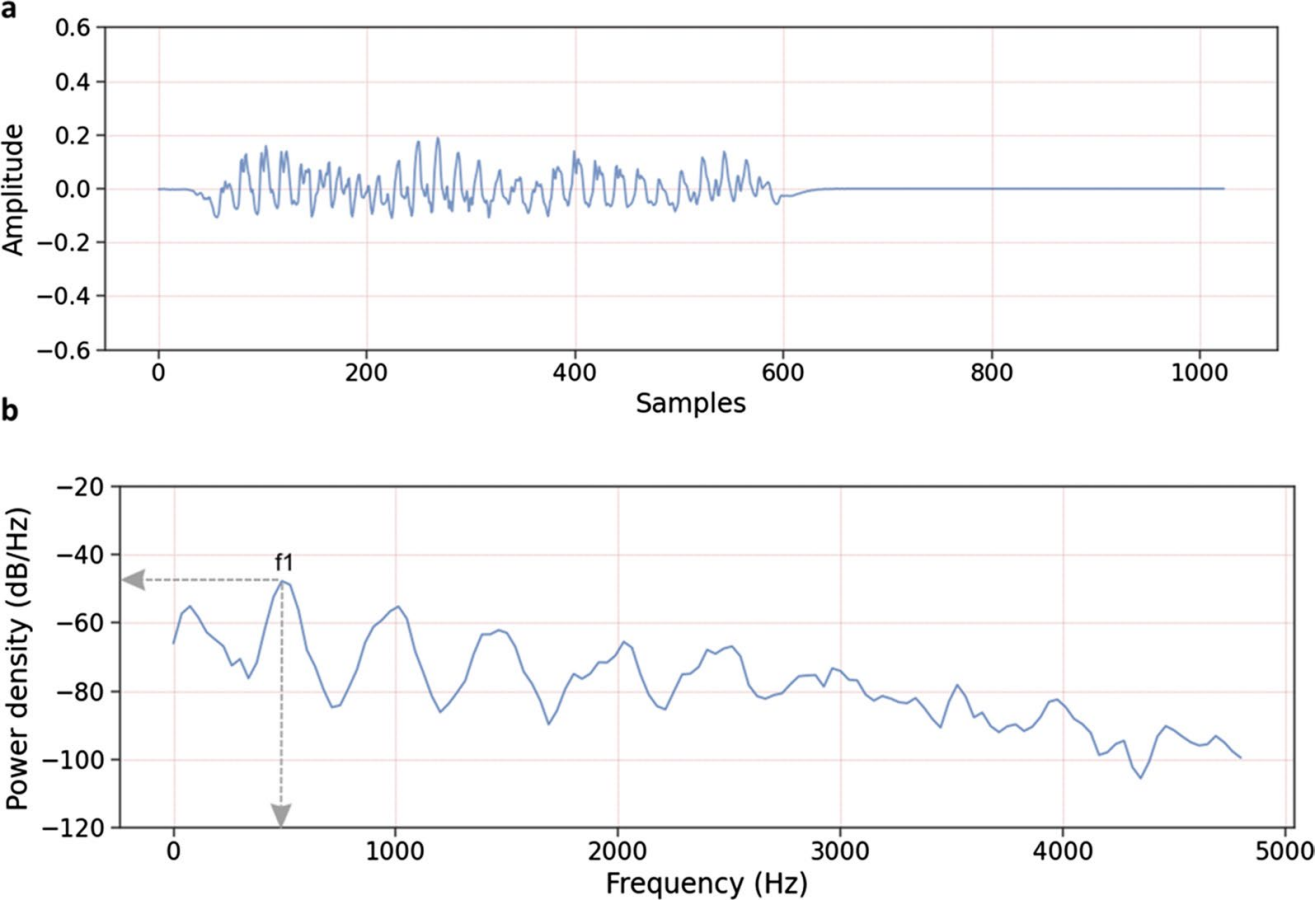
**a** Example of a recorded mosquito flight with ADC sample number (0 to 1023) on the *x*-axis and amplitude on the *y*-axis, scaled to a range of [− 1, 1], which equates to the full-scale range of the ADC. A high pass filter in the optical receiver attenuates frequencies < 300 Hz to remove electronic offsets and low-frequency noise, which also attenuates the signal due to the body of the insect. Baseline correction has been applied by subtracting the average value of the recording from each data point in the recording. **b** Power spectral density (PSD) plot of a typical mosquito flight. The wingbeat fundamental peak is labelled as f1. The fundamental frequency is indicated by the vertical arrow and the fundamental peak power by the horizontal arrow. The various peaks to the right of f1 are harmonics of f1, i.e. at frequencies of 2*f1, 3*f1, etc. The power density has units of (units^2^/Hz) on a logarithmic (dB) scale. A level of 0 dB/Hz corresponds to a white noise signal time domain signal with a power density of 1.0 unit^2^/Hz. The fundamental peak power density levels in this study are typically < − 40 dB/Hz, i.e. < 1 × 10^–4^ units^2^/Hz. The noise floor of the system, i.e. with sensor active but with no insect in the sensing zone, is < − 85 dB/Hz from 0 to 300 Hz and < − 90 dB/Hz from 300 Hz

Each recording corresponds to a different mosquito, i.e. trapped mosquitoes were not re-used to generate more recordings. Wingbeat files were tagged with species and sex class by the operator. After each experiment, the wingbeat recordings were downloaded from the sensor and processed using a Python script to produce playable and viewable audio files, as depicted in Fig. 2a. Wingbeat recordings were examined manually, and those deemed to be invalid, such as recordings containing more than one mosquito or where a mosquito may have hit the wall of the flight tube, were excluded from the dataset. The excluded recordings represented 2.3% of the data.

The resulting dataset contained 4335 wingbeat recordings, comprising 2472 of *Aedes* genus (882 *Ae. aegypti* and 1590 *Ae. albopictus*) and 1863 of *Culex* genus (all *Cx. pipiens*). There were 1211 *Aedes* females, 1261 *Aedes* males, 964 *Culex* females and 899 *Culex* males. Females were in an age range of 2 to 16 days old and males were in an age range of 2 to 9 days old. These age ranges provide a representative variety in the dataset.

All recordings took place with the sensor and trap located in the laboratory facilities of IRTA-CReSA during daylight hours. The average ambient temperature measured was 25.8 (standard deviation = 1.2 °C).

### Feature extraction

The following five features were extracted from each wingbeat recording via the application of digital signal processing methods:

- The power spectral density (PSD) shows the power of the signal at different frequencies. It is calculated using Welch’s method [44], in which the wingbeat recording is divided into several overlapping segments. A windowing function is applied to each of the segments and a series of periodograms is obtained by calculating the power spectrum of each windowed segment. Finally, the periodograms are averaged to give the PSD [45]. A PSD plot of a typical mosquito recording is shown in Fig. 2b.
- Wingbeat fundamental frequency in Hertz (Hz) is determined from the PSD as shown in Fig. 2b using a peak search method. The wingbeat fundamental frequency is the frequency at which a mosquito flaps its wings. It is characteristic of mosquito taxonomy and sex and varies depending on intrinsic variables of mosquito biology (size, age, parity status, mating behaviour) [16, 35, 36, 38] and environmental variables such as temperature [37]. The typical range of mosquito wingbeat fundamental frequencies is 300 to 900 Hz [40].
- The fundamental peak power density (dB/Hz) (hereafter referred to as fundamental peak power) is also determined from the PSD as shown in Fig. 2b and represents the peak power density of the sensor output at the wingbeat fundamental frequency. It is equivalent to the intensity of the sound produced by a flying mosquito, typically ranging from 40 to 80 dB [46, 47].
- The spectrogram is a series of spectra calculated from multiple overlapping segments of the wingbeat recording. Each spectrum is generated by applying a Fourier transform to the segment to provide information about the amplitude of the various frequency components in the segment. The spectrogram represents the variations of the frequency content of the signal over time rather than an average for the whole signal as given by the PSD [48].
- Mel Frequency Cepstral Coefficients (MFCCs) are calculated by converting the frequencies of a spectrogram to the Mel scale and applying overlapping triangular filter banks before calculating the cepstrum by transforming the spectra to a logarithmic scale and then applying an inverse Fourier transform [49]. Please refer to Additional file 1: Text S1 and Fig. S1 for further details.

The PSDs have 257 values, generated using a window length of 512 samples. The spectrograms and MFCCs are obtained using nine segments of 512 samples; then, 16 Mel filter banks are applied to each spectrum to give a total of 144 values. All the MFCC coefficients are used.

Each individual feature and one combined feature (fundamental frequency and fundamental peak power) were used for the machine learning models.

**Fig. 3:**
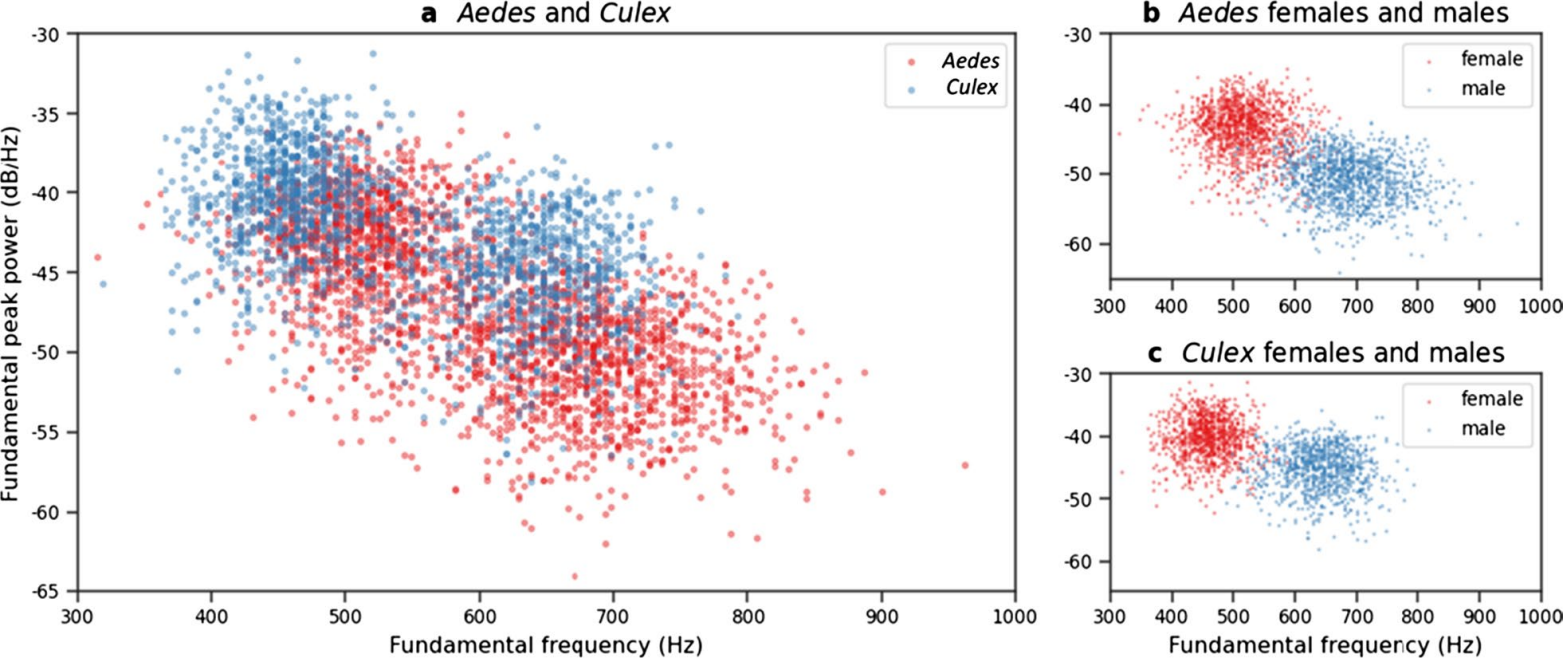
**a** Scatterplot of wingbeat fundamental frequency and peak power for the full dataset showing *Aedes* genus in red and *Culex* in blue. **b** Scatter plot of wingbeat fundamental frequency and peak power for *Aedes* genus showing females in red and males in blue. **c** Scatter plot of wingbeat fundamental frequency and peak power for *Culex* genus showing females in red and males in blue

A scatter plot of the wingbeat fundamental frequency and peak power features is shown in Fig. 3a for the entire dataset, in Fig. 3b for all *Aedes* samples and in Fig. 3c for all *Culex* samples. In Fig. 3a, which is coloured by genus, a high degree of overlap between the genera is observed. In Fig. 3b and c, which are coloured by sex, two clearly separated clusters are observed. The distributions of the two single-value features, fundamental frequency and fundamental peak power, for the three classifications are shown in Additional file 1: Fig. S2.

### Machine learning

The goal of the machine learning process was to compare the performance of five selected machine learning algorithms using the features described above, in classifying mosquito genus and sex. A labelled dataset consisting of the feature set was used to train, evaluate and compare the classification models. The following five machine learning algorithms were used: logistic regression (LR), gradient boosting (GB), random forests (RF), support vector machines (SVM) and a fully connected deep neural network (DNN). These algorithms were chosen because of their widespread usage and good performance [50]. A brief overview of each algorithm is given in Additional file 1: Text S2. Of these algorithms, the more complex ones, such as DNN or RF, were also used with the single-value features (fundamental frequency and fundamental peak power) because they can model non-linearities, unlike LR.

Three classification tasks were performed: one genus classification (*Aedes/Culex*) and two sex classifications (male/female), one for each genus (sex of *Aedes*, sex of *Culex*). The logic of the classification process is shown in Additional file 1: Fig. S3.

Balanced datasets, i.e. datasets that contained an equal number of samples in each class, were used to make an unbiased assessment. They were obtained by randomly under-sampling the classes which had a higher number of available samples.

Model performance was assessed using the accuracy metric, which is calculated by dividing the number of correct predictions by the total number of predictions. The accuracy metric is a simple evaluation metric, which makes it easy to interpret, and is appropriate when using balanced datasets.

The typical machine learning process consists of training, validation and testing. In the training phase, the model is fitted to the data with different configurations of the algorithm determined by hyperparameters, which can have a significant impact on performance. In the validation phase, the performances of the models trained with the different configurations are compared and the best one is selected. The testing phase assesses how well the model generalizes on previously unused data. A schematic overview of the training, validation and testing approach employed in this work is shown in Additional file 1: Fig. S4.

Seventy-five percent of the recordings in each dataset were chosen randomly to create a training set for use in the training and validation phase. Training and validation were done using fourfold cross-validation, in which the training set is split into four parts of equal size and the model being optimized is trained on three of the four parts and validated on the fourth part. This process is done four times using a different part of the training set for the cross-validation in each iteration. The final cross-validation score was obtained by averaging the four cross-validation results. The model with the best cross-validation score was then selected for testing.

The remaining 25% of each dataset, i.e. that part which was not allocated to training and validation, was used to test the performance of the trained model. Since the data in the test set are completely new to the model, accuracy results for the test set are an indication of how well the model generalizes on new data, and good results cannot be attributed to overfitting of the model.

Error analysis consists of analysing the training and validation accuracies obtained during the training and validation phase. If the training accuracy is considerably higher than the validation accuracy, it indicates overfitting, so more samples could help to improve the model. If, on the other hand, training and validation accuracies have a similar low score, it indicates that the model is too simple and that more training data would probably not help. In this case, the model could possibly be improved by using a different algorithm which is able to learn more complex relationships or to use more features.

Programming was done in *Python* [51]. For model generation, *scikit-learn* [52], *TensorFlow* [53] and *XGBoost* [54] were used. Regarding execution times, training of the models took days to weeks, but once done, each new sample was classified in under 1 s.

## Results

### Genus classification

In the genus classification, mosquitoes were classified into *Aedes* and *Culex* genus. A total of 2688 samples were used comprising: 1344 *Aedes* (672 *Ae. albopictus* and 672 *Ae. aegypti*) and 1344 *Culex* (all *Cx. pipiens*) with an equal number of males and females for each species. The dataset was split 75%/25% into the training data set (2016 samples) and the test set (672 samples). The accuracy results for genus classification on the test set are shown in Table 1 with the best performing algorithm for each feature shown in bold. The best result for genus classification was obtained for the DNN algorithm trained on the spectrogram feature, with an accuracy of 94.2%.

**Table 1:** Accuracy results for genus classification with best results per feature indicated by a superscript letter

Feature	Algorithm				
	LR (%)	GB (%)	RF (%)	SVM (%)	DNN (%)
Fundamental frequency	55.2	67.3^a^	65.9	65.5	66.1
Fundamental peak power	68.9	70.1^a^	69.6	69.8	70.0
Fundamental frequency and peak power	70.1	77.7	77.2	77.2	77.8^a^
PSD	84.8	92.3^a^	89.0	90.5	90.3
Spectrogram	90.5	93.2	91.2	93.4	94.2^a^
MFCC	89.3	93.2^a^	90.2	93.0	93.2^a^

### Sex classification of *Aedes*

In this classification, mosquitoes of the *Aedes* genus were classified into males and females. A total of 1344 samples were used, comprising 672 females and 672 males, with each sex group comprising 336 *Ae. aegypti* and 336 *Ae. albopictus*. The dataset was split 75%/25% into the training data set (1008 samples) and the test set (336 samples). The results for this classification on the test set are shown in Table 2. The best performing algorithms for sex classification of *Aedes* were logistic regression trained on spectrogram and MFCC, and gradient boosting trained on MFCC, with an accuracy of 99.4% in each case.

**Table 2:** Accuracy results for sex classification of *Aedes* with best results per feature indicated by a superscript letter

Feature	Algorithm				
	LR (%)	GB (%)	RF (%)	SVM (%)	DNN (%)
Fundamental frequency	95.5	95.5	95.5	95.5	95.5
Fundamental peak power	86.9	89.5^a^	89.5	89.2	89.3
Fundamental frequency and peak power	98.2	96.7	97.0	98.5	97.9^a^
PSD	97.0	98.8^a^	97.9	98.8^a^	98.2
Spectrogram	99.4^a^	98.8	98.8	99.1	98.8
MFCC	99.4^a^	99.4^a^	98.8	98.8	98.8

### Sex classification of *Culex*

In this classification, mosquitoes of the *Culex* genus (all *Cx. pipiens*) were separated into males and females. A total of 1560 samples were used comprising 780 females and 780 males. The dataset was split 75%/25% into the training data set (1170 samples) and the test set (390 samples). The results for this classification on the test set are shown in Table 3. For *Culex* sex classification, an accuracy of 100% was achieved by all five algorithms trained on MFCC; by logistic regression, SVM and DNN trained on spectrogram; and by SVM trained on PSD.

**Table 3:** Accuracy results for sex classification of *Culex* with best results per feature indicated by a superscript letter

Feature	Algorithm				
	LR (%)	GB (%)	RF (%)	SVM (%)	DNN (%)
Fundamental frequency	98.0	98.0	98.0	98.0	98.0
Fundamental peak power	83.4	81.3	81.5	83.1	83.6^a^
Fundamental frequency and peak power	98.7^a^	98.7^a^	98.5	98.7^a^	98.7^a^
PSD	99.7	99.2	99.2	100^a^	99.7
Spectrogram	100^a^	99.7	99.7	100^a^	100^a^
MFCC	100^a^	100^a^	100^a^	100^a^	100^a^

### Summary of the best classification results

A summary of the classification results, which includes the best performing algorithms and features for each classification, is given in Table 4 in which training and validation accuracies are also listed, with an indication of how the results might be improved. The corresponding hyperparameters are listed in Additional file 1: Table S1.

**Table 4:** Summary of machine learning classification results

Classification task	Using the test set			Using the training dataset			Error analysis indication
	Best test accuracy (%)	Best feature	Best algorithm	No. of samples	Training accuracy (%)	Validation accuracy (%)	
Genus	94.2	Spectrogram	DNN	2016	100	95	Slight overfitting: more training samples
Sex *Aedes*	99.4	Spectrogram	LR	1008	99.5	99.5	No overfitting
		MFCC	LR, GB				
Sex *Culex*	100	PSD	SVM	1170	100	100	No error
		Spectrogram	LR, SVM, DNN				
		MFCC	All algorithms				

The best accuracy results were 94.2% for genus classification, 99.4% for sex classification of *Aedes* and 100% for sex classification of *Culex*.

For genus, the training accuracy was 100% and the cross-validation accuracy was significantly lower (95%), which indicates that the model overfits slightly and its performance could possibly be improved with more training samples.

For *Aedes* sex classification, although the best models gave a near perfect accuracy, the training accuracy and cross-validation accuracy are similar (99.5%), which indicates that the model could possibly be improved with a more complex algorithms and/or features rather than with more training samples. In case of *Culex* sex classification the accuracy was 100%, so no error analysis was necessary.

## Discussion

In the present study, 4335 mosquito flights were recorded using a novel optical sensor. The sensor was attached to the entrance of a commercial mosquito suction trap inside an insect rearing cage, with mosquitoes flying freely within the cage until they were sucked in by the trap, through the sensor and into the catch bag within the trap. Each flight recording made by the sensor corresponded to a different mosquito. Five features were extracted from each recording and used with five different machine learning algorithms for classification of mosquito genus and sex.

One of the features used was wingbeat fundamental frequency, which has been used in many studies for insect characterization and classification [15, 16, 23, 25, 35, 38, 55]. Differences in reported values of wingbeat frequency between studies can be due to intrinsic and/or extrinsic variables such as size, parity status, age and ambient conditions [16, 34, 36, 42, 46]. In this study, the wingbeat fundamental frequency feature gave a high accuracy in sex classification in both *Aedes* (95.5%) and *Culex* (98%), but it scored lower (67.3%) in genus classification. These results are consistent with the fundamental frequency histograms in Additional file 1: Fig. S2, which show very little overlap between the distributions of males and females, especially for *Culex* (Additional file 1: Fig. S2c) and considerable overlap between genera (Additional file 1: Fig. S2a). In the fundamental peak power histograms of Additional file 1: Fig. S2b, c, a higher degree of overlap is observed between the distributions of males and females, especially for *Culex*, which helps explain why the accuracy for sex using this feature alone (89.5% for *Aedes* and 83.6% for *Culex*) was lower than that of fundamental frequency alone.

As other studies have indicated [16, 18, 39, 40], the use of the wingbeat frequency alone as a feature to differentiate between taxonomical classes or other attributes of mosquito biology can be challenging because of overlap in wingbeat frequency distributions. To address this, other authors have used additional features (i.e. depolarization ratio) [16] or metadata (i.e. localization, environmental variables and circadian rhythm) [18] in combination with fundamental frequency to improve their classification methods. In the present work, we have tested several features apart from or in combination with the fundamental frequency to better classify mosquito genus and sex.

The use of both fundamental frequency and fundamental peak power yielded better performance in sex and genus classification than fundamental frequency alone. Although the effect of signal intensity or power has been investigated in mosquito mating and courtship behavioural experiments [46, 47], to the best of our knowledge, fundamental peak power has not been used as a feature in mosquito classification studies. In other sensor systems, the reported signal intensity or power may depend on the position and orientation of the flying mosquito with respect to the sensor [56], whilst our optical setup was designed to measure wingbeat power relatively independently of the position and orientation of the mosquito within the sensing volume.

Despite the better results obtained in this work using the fundamental frequency and power features compared with fundamental frequency alone, the more complex spectrogram and MFCC features provided the best performance for genus and sex classification. MFCCs are normally used in applications such as speech recognition [57] or music information retrieval [58], and although MFCCs are based on human perception of pitch, they have given good results in sound recognition studies with mosquitoes and other insects [34, 49, 59, 60].

In this study, the best performing machine learning algorithm depended on the classification task. For genus classification, DNN showed the best performance, with an accuracy of 94.2%, trained on the spectrogram feature. In another work [33], DNN also gave the best performance for genus classification between *Aedes* and *Culex*. For sex classification, the best performing algorithms and features were LR with spectrogram or MFCC and GB with MFCC. Different machine learning algorithms were also compared for mosquito classification in a previous study [17], and it was concluded that the best algorithm for complex classification tasks was SVM. In our study, SVM had an accuracy of 93.4% for genus, although DNN, which was not studied in [17], performed slightly better (94.2%). The classification of mosquito genus achieved a high accuracy of 94.2% while the classification of sex achieved 99.4% and 100% for *Aedes* and *Culex* respectively. The training and validation accuracies indicate that genus classification could possibly be improved with more training samples.

Other studies have successfully achieved automatic classification of genus [25, 33] and sex [16, 24] using machine learning with relatively large datasets [34] and placing emphasis on class balance [17]. However, only a small number of sensor studies have been performed using a mosquito suction trap, either without an automatic classification system [39] or with only mosquito and non-mosquito counting and without differentiating mosquito genus and sex [61]. To our knowledge, we present the first sensor system for use with a commercial mosquito suction trap, which provides automatic classification of genus and sex with high performance, based on a large number of training samples, with class balance. Planned further work includes the study of species classification, study of age groups, training of models with more features and feature combinations, and testing of the system in the field.

## Conclusions

In this work, we have presented the results of a novel sensor system for genus and sex classification of *Aedes* and *Culex* mosquitoes captured by a commercial suction trap in laboratory conditions. The obtained results are encouraging for the use of the sensor with standard suction traps in the field, for the remote surveillance and classification of genus and sex of *Aedes* and *Culex* mosquitoes.

## Supplementary Information

**Additional file 1: Text S1.** Mel spectrogram and MFCC generation process. **Figure S1.** Diagram to illustrate MFCC generation. **Figure S2.** Histogram plots showing the distributions of fundamental frequency (top) and fundamental peak power (bottom) for **a** Genus, **b** *Aedes* sex and **c** *Culex* sex. **Text S2.** Description of the machine learning algorithms used in this work. **Figure S3.** Representation of the machine learning classifications (in bold text), with their respective classes immediately below and indicated by the arrow heads. **Figure S4.** Schematic overview of the training, validation and testing approach. **1** Dataset is randomly separated into training and test sets, accounting for 75% and 25% of the whole dataset respectively. **2** Training set is separated using fourfold cross-validation into four folds with an equal number of samples in each fold. **3** Four iterations of training and validation take place using a different fold for validation in each iteration. **4** Model with best average validation score, obtained by averaging the four cross-validation results, is selected. **5** Model is evaluated using test set (containing data which was previously unused) to obtain test score. **Table S1.** Hyperparameters of the trained models which achieved the highest accuracies.

## Data Availability

The datasets generated during and/or analysed during the current study are not publicly available due to the protection of intellectual property defined under the H2020 agreement no. 853758, but are available from the corresponding author on reasonable request.

## Abbreviations

2D: Two-dimensional
ADC: Analog to digital
DNN: Dense neural network
GB: Gradient boosting
IoT: Internet of things
LED: Light-emitting diodes
LR: Logistic regression
MFCC: Mel Frequency Cepstral Coefficients
PSD: Power spectral density
RF: Random forest
SVM: Support vector machines
VBD: Vector-borne disease
