# Supplementary material for: A novel optical sensor system for the automatic classification of mosquitoes by genus and sex with high levels of accuracy
Source: Parasit Vectors. 2022 Jun 6;15:190. doi: 10.1186/s13071-022-05324-5 (PMC9169302; doi:10.1186/s13071-022-05324-5)
Supplement: Supplementary file 1 — Additional file 1: Text S1. Mel spectrogram and MFCC generation process. Figure S1. Diagram to illustrate MFCC generation. Figure S2. Histogram plots showing the distributions of fundamental frequency (top) and fundamental peak power (bottom) for a Genus, b Aedes sex and c Culex sex. Text S2. Description of the machine learning algorithms used in this work. Figure S3. Representation of the machine learning classifications (in bold text), with their respective classes immediately below and indicated by the arrow heads. Figure S4. Schematic overview of the training, validation and testing approach. 1 Dataset is randomly separated into training and test sets, accounting for 75% and 25% of the whole dataset respectively. 2 Training set is separated using fourfold cross-validation into four folds with an equal number of samples in each fold. 3 Four iterations of training and validation take place using a different fold for validation in each iteration. 4 Model with best average validation score, obtained by averaging the four cross-validation results, is selected. 5 Model is evaluated using test set (containing data which was previously unused) to obtain test score. Table S1. Hyperparameters of the trained models which achieved the highest accuracies. [file 13071_2022_5324_MOESM1_ESM.docx]

**Additional file 1**

**Text S1.** Mel spectrogram and MFCC generation process.

The Mel scale is a perceptual scale which relates sound frequency in Hertz to the pitch perceived by the human auditory system [1].

For the filter banks, the minimum frequency is taken as 0 Hz and the maximum frequency is taken as the Nyquist frequency of the sampling system, i.e., $\frac{9603 Hz}{2}=4801.5 Hz$, and these are converted from Hertz ($f$) to Mel ($m$) using the following formula [2]:

$$m\left( f \right)=1125ln\left( 1+\frac{f}{700} \right)$$

This span (in Mels) is divided into “n” bands of equal width where “n” is the number of filters applied, and the end points of each filter are converted to Hertz using the following formula:

$$f=700\left( {10}^{\text{m/2595}}-1 \right)$$

The resulting values, form the base of each triangular filter in the frequency domain in which the base of each triangle is at zero and the peak is at one, giving values between zero and one along the legs of each triangle. Within each filter band, the values of the frequency spectra are multiplied by the corresponding value of the leg of the triangle and the results are then summed to give one value of the Mel spectrum. As such, the Mel spectrum consists of “n” values. Each Mel spectrum is converted to a logarithmic scale and joined to form a Mel spectrogram [3]. An inverse Fourier transform is then applied to generate the MFCC.

The process of generating the MFCC feature is represented in Fig. S1.

**Fig. S1.** Diagram to illustrate MFCC generation


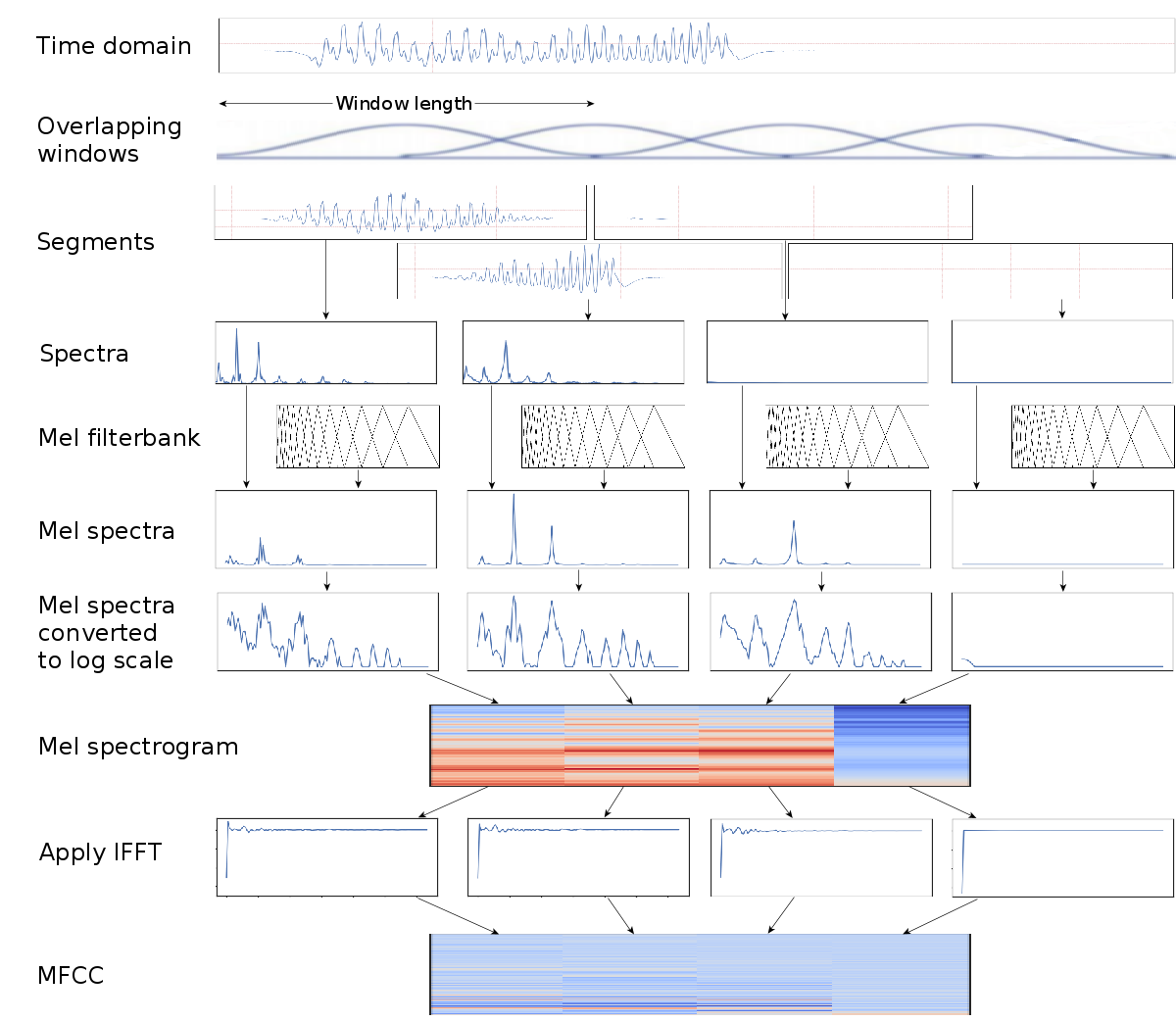


**Fig. S2.** Histogram plots showing the distributions of fundamental frequency (top) and fundamental peak power (bottom) for **a** Genus, **b** *Aedes* sex, and **c** *Culex* sex.


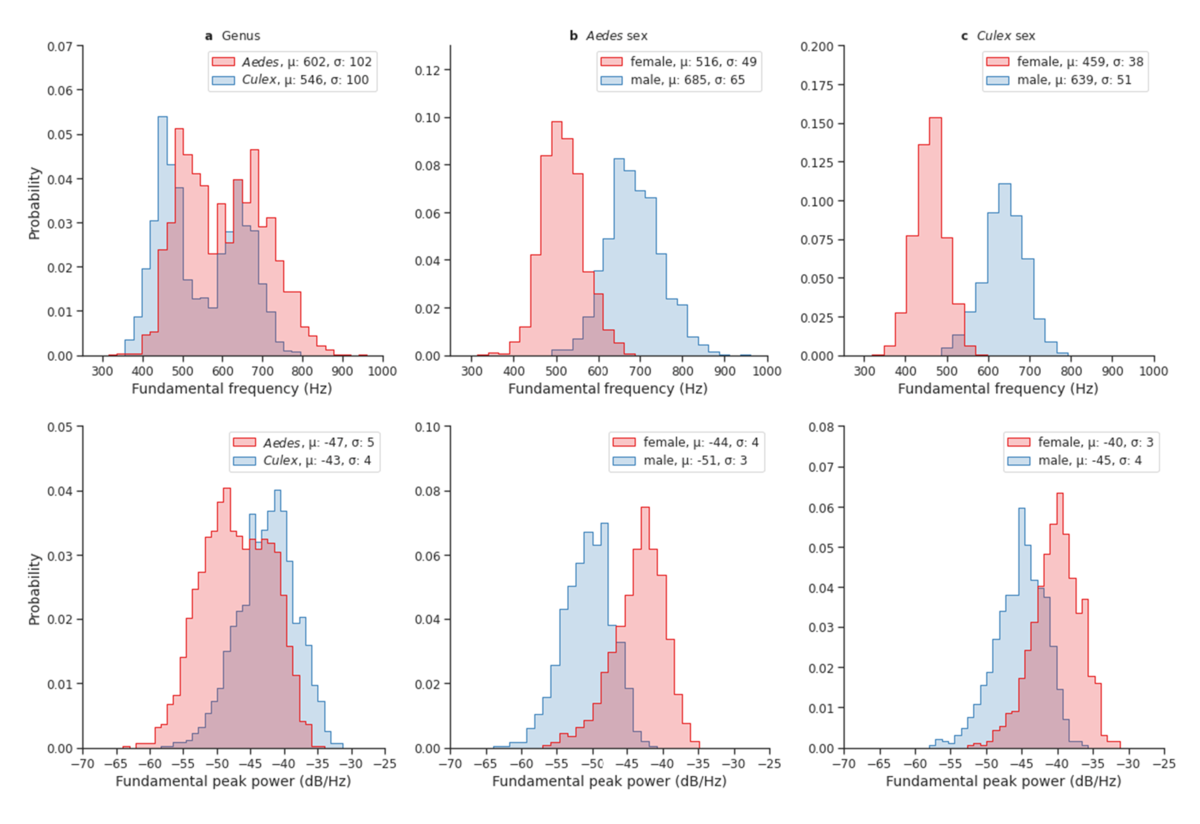


**Text S2.** Description of the machine learning algorithms used in this work.

The following provides a brief description of the machine learning algorithms employed in this work.

- LR fits a sigmoid function to the observations (predictor variables and labels) to generate a classification model [4, 5].
- GB uses the decision trees ensemble model which consists of different classification and regression trees (CART). In GB, the ensemble building is done incrementally so that each new tree helps correct the error of the previous tree [6, 7]. The optimized *XGBoost* library was used [8].
- RF also uses the decision tree ensemble method, but it grows the different trees simultaneously from a randomly chosen subset of the predictor variables and then they vote on the outcome [9]. Another method of randomization used in RF is bootstrapping where a subset of the samples of the training set is randomly chosen for each tree [10] which allows for non-linear models when using one or more predictor variables.
- SVM works by plotting the training samples in an n-dimensional space (where n is the number feature values used) and generating a hyperplane or hyperline that separates the different classes. The best hyperplane is chosen by maximizing the distance of the hyperplane to the closest samples of either class. These samples form the support vectors. For non-linear models, different kernel functions exist which transform non-linear spaces into linear-spaces[11].
- DNNs mimic the structure of the human brain and consist of multiple layers of relatively simple processing units that receive input and give output. The use of multiple layers and non-linear activation functions allows for non-linearity between predictor variables and outputs [12, 13].

The logic of the machine learning classification process is shown in Fig. S3.

**Fig. S3.** Representation of the machine learning classifications (in bold text), with their respective classes immediately below and indicated by the arrow heads.


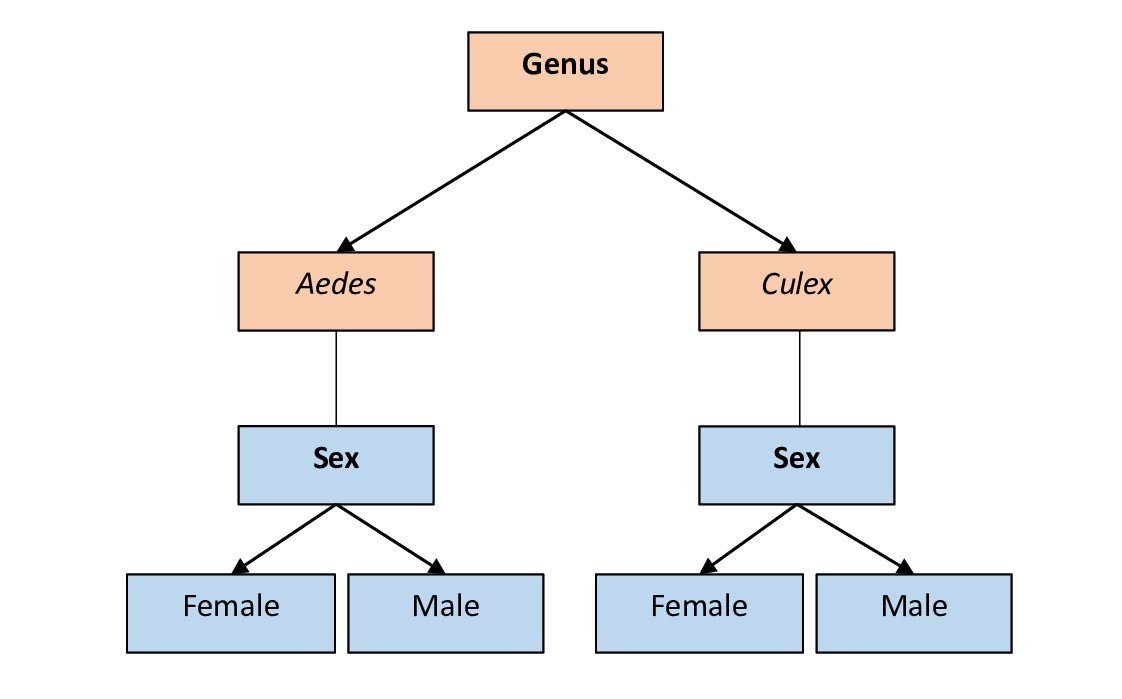


**Fig. S4.** Schematic overview of the training, validation and testing approach. **1** Dataset is randomly separated into training and test sets, accounting for 75% and 25% of the whole dataset respectively. **2** Training set is separated using 4-fold cross-validation into four folds with an equal number of samples in each fold. **3** Four iterations of training and validation take place using a different fold for validation in each iteration. **4** Model with best average validation score, obtained by averaging the four cross-validation results, is selected. **5** Model is evaluated using test set (containing data which was previously unused) to obtain test score.


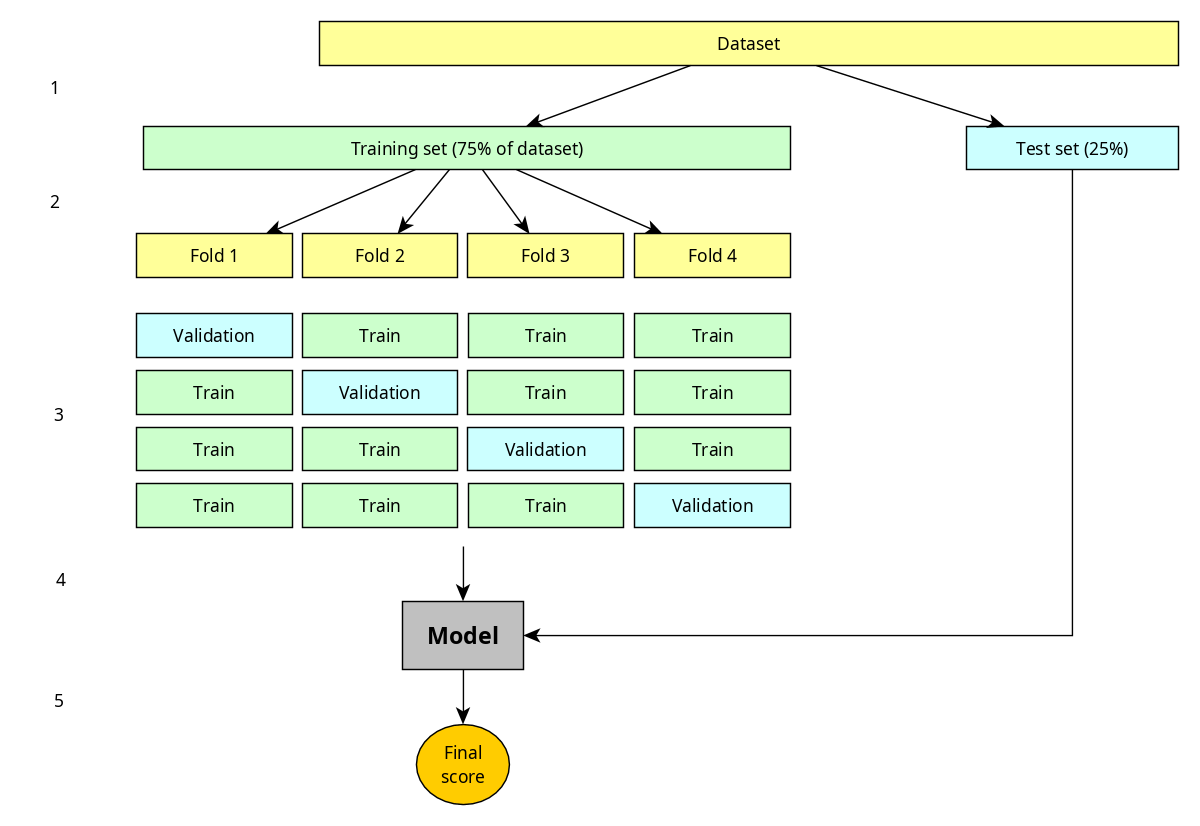


**Table S1.** Hyperparameters of the trained models which achieved the highest accuracies.

| **Classification task** | **Best feature** | **Best algorithm** | **Hyperparameters** |
| --- | --- | --- | --- |
| Genus | Spectrogram | DNN | layers: 4  epochs: 1400 |
| Sex *Aedes* | Spectrogram | LR | penalty: L2  C: 2.8 |
|  | MFCC | LR | penalty: L2,  C: 1.0 |
|  |  | GB | estimators: 200 |
| Sex *Culex* | PSD | SVM | kernel: RBF  C: 2.8 |
|  | Spectrogram | LR | penalty: L2  C: 1.0 |
|  |  | SVM | kernel: RBF  C: 1.0 |
|  |  | DNN | layers: 3 epochs: 1500 |
|  | MFCC | LR | penalty: L2  C: 1.0 |
|  |  | GB | estimators: 450 |
|  |  | RF | estimators: 100 |
|  |  | SVM | kernel: RBF  C: 1.0 |
|  |  | DNN | layers: 3  epochs:1500 |

References

1. Volkmann J, Stevens SS, Newman EB. A scale for the measurement of the psychological magnitude pitch. J Acoust Soc Am. 1937;doi:[10.1121/1.1901999](https://doi.org/10.1121/1.1901999).
2. Arias-Vergara T, Klumpp P, Vasquez-Correa JC, Nöth E, Orozco-Arroyave JR, Schuster M. Multi-channel spectrograms for speech processing applications using deep learning methods. Pattern Anal Applic. 2021;24:423-31.
3. Virtanen T, Vincent E, Gannot S. Time-frequency processing - Spectral properties. Wiley; 2018. Audio source separation and speech enhancement. Wiley. 2018. <https://hal.inria.fr/hal-01881426> Accessed 18 Sep 2021.
4. Peng CYJ, Lee KL, Ingersoll GM. An introduction to logistic regression analysis and reporting. J Educ Res. 2002;doi:[10.1080/00220670209598786](https://doi.org/10.1080/00220670209598786).
5. Kleinbaum DG, Klein M. Logistic regression: a self-learning text. Springer, 2010.
6. Friedman JH. Greedy function approximation: a gradient boosting machine. Ann Stat. 2001;29:1189-232.
7. Chen T, Guestrin C. XGBoost: a scalable tree boosting system. In: Proceedings of the 22nd ACM SIGKDD [International Conference on Knowledge Discovery and Data Mining](https://dl.acm.org/doi/proceedings/10.1145/2939672). San Francisco, California, USA. 2016 Aug 13-17. Association for computing machinery. 2016;doi:[10.1145/2939672.2939785](http://dx.doi.org/10.1145/2939672.2939785).
8. XGBoost. XGBoost documentation. <https://xgboost.readthedocs.io> Accessed 15 Sep 2021.
9. Breiman L. Random forests. Mach Learn. 2001;45:5-32.
10. Lee TH, Ullah A, Wang R. Bootstrap aggregating and random forest. Adv. Stud. Theor. Phys. 2020;doi:10.1007/978-3-030-31150-6_13.
11. Burges CJC. A tutorial on support vector machines for pattern recognition. Data Min. Knowl. Discov. 1998;2:121-67.
12. Schmidhuber J. Deep Learning in Neural Networks: an overview. Neural Netw. 2015; 61:85-117.
13. Zhang Z. A gentle introduction to artificial neural networks. [Ann Transl Med.](https://www.ncbi.nlm.nih.gov/pmc/articles/PMC5075856/) 2016; 4:370.
